# Dr. Lewis B. Andrews

**Published:** 1904-07

**Authors:** 


DR. LEWIS B. ANDREWS, of Rochester, N. Y., a graduate of the University of Buffalo, 1885, died at his home of heart disease, May 22, 1904, aged 46 years.

